# 3-Acetyl-1-(2-methylphenyl)thiourea

**DOI:** 10.1107/S1600536812011658

**Published:** 2012-03-24

**Authors:** Durre Shahwar, M. Nawaz Tahir, Muhammad Mansha Chohan, Naeem Ahmad, M. Asam Raza

**Affiliations:** a Department of Chemistry, Government College University, Lahore, Pakistan; b University of Sargodha, Department of Physics, Sargodha, Pakistan; c Department of Chemistry, University of Gujrat, Gujrat, Pakistan

## Abstract

In the title compound, C_10_H_12_N_2_OS, the toluene and the *N*-carbamothio­ylacetamide units are oriented at dihedral angle of 78.75 (5)°. An intra­molecular N—H⋯O hydrogen bond generates an *S*(6) ring. In the crystal, mol­ecules are linked into [101] chains by pairs of N—H⋯S hydrogen bonds [which generate *R*_2_^2^(8) loops] and pairs of O—H⋯O hydrogen bonds [which generate *R*_2_^2^(4) loops]. The two motifs alternate in the chain.

## Related literature

For related structures, see: Shahwar *et al.* (2012 ▶). For graph–set notation, see: Bernstein *et al.* (1995 ▶).
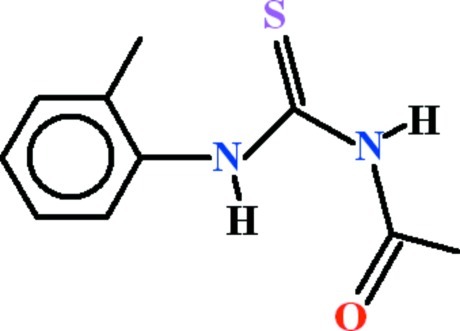


## Experimental

### 

#### Crystal data

C_10_H_12_N_2_OS
*M* *_r_* = 208.28
Monoclinic, 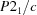

*a* = 5.0444 (2) Å
*b* = 20.7019 (9) Å
*c* = 9.9464 (4) Å
β = 95.116 (2)°
*V* = 1034.55 (7) Å^3^
*Z* = 4
Mo *K*α radiation
μ = 0.28 mm^−1^
*T* = 296 K
0.35 × 0.15 × 0.13 mm

#### Data collection

Bruker Kappa APEXII CCD diffractometer
Absorption correction: multi-scan (*SADABS*; Bruker, 2005 ▶) *T*_min_ = 0.915, *T*_max_ = 0.938
7696 measured reflections
1812 independent reflections
1512 reflections with *I* > 2σ(*I*)
*R*_int_ = 0.028

#### Refinement

*R*[*F* ^2^ > 2σ(*F* ^2^)] = 0.037
*wR*(*F* ^2^) = 0.088
*S* = 1.17
1812 reflections
129 parameters
H-atom parameters constrained
Δρ_max_ = 0.18 e Å^−3^
Δρ_min_ = −0.20 e Å^−3^

### 

Data collection: *APEX2* (Bruker, 2009 ▶); cell refinement: *SAINT* (Bruker, 2009 ▶); data reduction: *SAINT*; program(s) used to solve structure: *SHELXS97* (Sheldrick, 2008 ▶); program(s) used to refine structure: *SHELXL97* (Sheldrick, 2008 ▶); molecular graphics: *ORTEP-3 for Windows* (Farrugia, 1997 ▶) and *PLATON* (Spek, 2009 ▶); software used to prepare material for publication: *WinGX* (Farrugia, 1999 ▶) and *PLATON*.

## Supplementary Material

Crystal structure: contains datablock(s) global, I. DOI: 10.1107/S1600536812011658/hb6692sup1.cif

Structure factors: contains datablock(s) I. DOI: 10.1107/S1600536812011658/hb6692Isup2.hkl

Supplementary material file. DOI: 10.1107/S1600536812011658/hb6692Isup3.cml

Additional supplementary materials:  crystallographic information; 3D view; checkCIF report

## Figures and Tables

**Table 1:** Hydrogen-bond geometry (Å, °)

*D*—H⋯*A*	*D*—H	H⋯*A*	*D*⋯*A*	*D*—H⋯*A*
N1—H1⋯O1	0.86	1.99	2.664 (2)	135
N1—H1⋯O1^i^	0.86	2.50	3.172 (2)	135
N2—H2⋯S1^ii^	0.86	2.52	3.3747 (17)	171

Symmetry codes: (i) 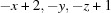; (ii) 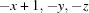.

**Fig. 1.**
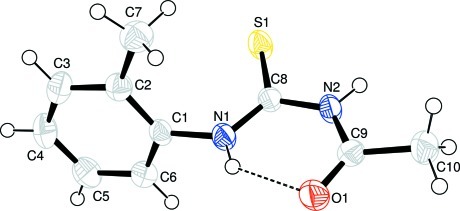
View of the title compound with displacement ellipsoids are drawn at the 50% probability level. The dotted lines represent the intra-molecular H-bondings.

**Fig. 2.**
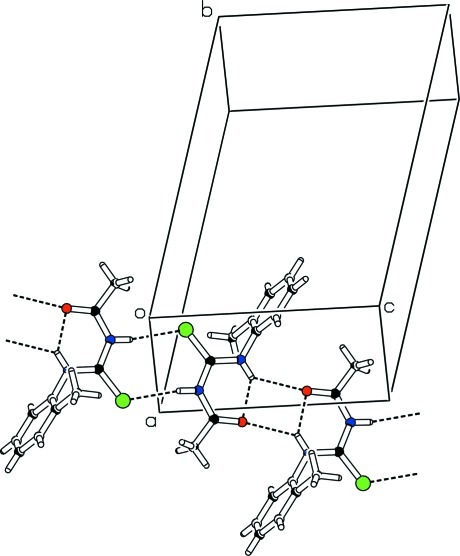
The partial packing (PLATON; Spek, 2009) which shows that molecules form polymeric chains extending along [1 0 1] direction.

## supplementary crystallographic information

### Comment

The title compound I (Fig. 1) has been synthesized in continuation of our efforts to find new enzyme inhibitors.

The crystal structures of *N*-(phenylcarbamothioyl)acetamide (Shahwar *et al.*, 2012) has been published which is related to the title compound (I).

In (I), the toluene group A (C1–C7) and the *N*-carbamothioylacetamide moiety B (N1/C8/S1/N2/C9/O1/C10) are planar with r. m. s. deviation of 0.0058 Å and 0.0278 Å, respectively. The dihedral angle between A/B is 78.75 (5)°. There exist classical intramolecular H–bonding of N—H···O type (Table 1, Fig. 1) with *S*(6) ring motif (Bernstein *et al.*, 1995). The molecules are dimerized due to N—H···S type of hydrogen bonds with *R*_2_^2^(8) ring motifs (Table 1, Fig. 2). The dimers are interlinked from S(6) ring motifs due to strong N—H···O H–bondings (Table 1, Fig. 2) with centrosymmetric four membered ring motif (—O···H···O···H···O—) (Table 1, Fig. 2). The polymeric chains extend along the base vector [101].

### Experimental

The title compound (I) was synthesized by adding (0.1 mol, 7.13 ml) of acetylchloride dropwise to a stirred solution of KSCN (0.11 mol) in dry acetone (50 ml), followed by slow addition of toluidine (0.1 mol) in dry acetone (25 ml). The mixture was refluxed for 5–10 min, then poured on ice cooled water, which resulted in crude precipitate. Recrystallization of the precipitate in ethylacetate yielded colourless needles.

### Refinement

The H-atoms were positioned geometrically (C—H = 0.93–0.96 Å, N—H = 0.86 Å) and refined as riding with *U*_iso_(H) = *xU*_eq_(C, N), where *x* = 1.5 for methyl groups and *x* = 1.2 for other H atoms.

### Crystal data

**Table:**

C_10_H_12_N_2_OS	*F*(000) = 440
*M**_r_* = 208.28	*D*_x_ = 1.337 Mg m^−^^3^
Monoclinic, *P*2_1_/*c*	Mo *K*α radiation, λ = 0.71073 Å
Hall symbol: -P 2ybc	Cell parameters from 1513 reflections
*a* = 5.0444 (2) Å	θ = 2.0–25.2°
*b* = 20.7019 (9) Å	µ = 0.28 mm^−^^1^
*c* = 9.9464 (4) Å	*T* = 296 K
β = 95.116 (2)°	Needle, white
*V* = 1034.55 (7) Å^3^	0.35 × 0.15 × 0.13 mm
*Z* = 4	

### Data collection

**Table:**

Bruker Kappa APEXII CCD diffractometer	1812 independent reflections
Radiation source: fine-focus sealed tube	1512 reflections with *I* > 2σ(*I*)
Graphite monochromator	*R*_int_ = 0.028
Detector resolution: 8.00 pixels mm^-1^	θ_max_ = 25.2°, θ_min_ = 2.0°
ω scans	*h* = −5→6
Absorption correction: multi-scan (*SADABS*; Bruker, 2005)	*k* = −24→22
*T*_min_ = 0.915, *T*_max_ = 0.938	*l* = −11→11
7696 measured reflections	

### Refinement

**Table:**

Refinement on *F*^2^	Primary atom site location: structure-invariant direct methods
Least-squares matrix: full	Secondary atom site location: difference Fourier map
*R*[*F*^2^ > 2σ(*F*^2^)] = 0.037	Hydrogen site location: inferred from neighbouring sites
*wR*(*F*^2^) = 0.088	H-atom parameters constrained
*S* = 1.17	*w* = 1/[σ^2^(*F*_o_^2^) + (0.0311*P*)^2^ + 0.355*P*] where *P* = (*F*_o_^2^ + 2*F*_c_^2^)/3
1812 reflections	(Δ/σ)_max_ < 0.001
129 parameters	Δρ_max_ = 0.18 e Å^−^^3^
0 restraints	Δρ_min_ = −0.20 e Å^−^^3^

### Special details

**Table:**

Geometry. Bond distances, angles *etc*. have been calculated using the rounded fractional coordinates. All su's are estimated from the variances of the (full) variance-covariance matrix. The cell e.s.d.'s are taken into account in the estimation of distances, angles and torsion angles
Refinement. Refinement of *F*^2^ against ALL reflections. The weighted *R*-factor *wR* and goodness of fit *S* are based on *F*^2^, conventional *R*-factors *R* are based on *F*, with *F* set to zero for negative *F*^2^. The threshold expression of *F*^2^ > σ(*F*^2^) is used only for calculating *R*-factors(gt) *etc*. and is not relevant to the choice of reflections for refinement. *R*-factors based on *F*^2^ are statistically about twice as large as those based on *F*, and *R*- factors based on ALL data will be even larger.

### Fractional atomic coordinates and isotropic or equivalent isotropic displacement parameters (Å^2^)

**Table:**

	*x*	*y*	*z*	*U*_iso_*/*U*_eq_	
S1	0.39407 (11)	0.07983 (3)	0.11730 (5)	0.0401 (2)	
O1	1.0674 (3)	−0.02473 (7)	0.36353 (14)	0.0456 (5)	
N1	0.7141 (3)	0.07120 (8)	0.34350 (16)	0.0337 (5)	
N2	0.7701 (3)	−0.00655 (8)	0.18163 (16)	0.0326 (5)	
C1	0.5997 (4)	0.12750 (10)	0.40024 (19)	0.0312 (6)	
C2	0.6773 (4)	0.18879 (10)	0.3631 (2)	0.0348 (7)	
C3	0.5651 (4)	0.24080 (11)	0.4260 (2)	0.0440 (8)	
C4	0.3876 (4)	0.23224 (12)	0.5219 (2)	0.0493 (8)	
C5	0.3150 (5)	0.17099 (12)	0.5564 (2)	0.0491 (8)	
C6	0.4207 (4)	0.11826 (11)	0.4958 (2)	0.0399 (7)	
C7	0.8746 (4)	0.19878 (12)	0.2608 (2)	0.0462 (8)	
C8	0.6379 (4)	0.04791 (9)	0.22219 (19)	0.0294 (6)	
C9	0.9799 (4)	−0.03869 (10)	0.2492 (2)	0.0334 (7)	
C10	1.0907 (4)	−0.09266 (11)	0.1711 (2)	0.0456 (8)	
H1	0.83956	0.05146	0.39124	0.0404*	
H2	0.71344	−0.02218	0.10425	0.0391*	
H3	0.61134	0.28255	0.40262	0.0528*	
H4	0.31710	0.26787	0.56314	0.0591*	
H5	0.19428	0.16508	0.62076	0.0589*	
H6	0.37200	0.07669	0.51911	0.0478*	
H7A	0.79640	0.18600	0.17331	0.0692*	
H7B	1.03029	0.17317	0.28474	0.0692*	
H7C	0.92326	0.24358	0.25886	0.0692*	
H10A	1.21650	−0.11680	0.22928	0.0684*	
H10B	1.17794	−0.07517	0.09732	0.0684*	
H10C	0.94861	−0.12060	0.13674	0.0684*	

### Atomic displacement parameters (Å^2^)

**Table:**

	*U*^11^	*U*^22^	*U*^33^	*U*^12^	*U*^13^	*U*^23^
S1	0.0437 (3)	0.0418 (3)	0.0328 (3)	0.0132 (3)	−0.0077 (2)	−0.0084 (3)
O1	0.0543 (9)	0.0469 (10)	0.0337 (9)	0.0164 (7)	−0.0069 (7)	−0.0033 (7)
N1	0.0381 (9)	0.0333 (10)	0.0284 (9)	0.0089 (7)	−0.0038 (7)	−0.0049 (7)
N2	0.0363 (9)	0.0318 (10)	0.0289 (9)	0.0056 (7)	−0.0007 (7)	−0.0084 (7)
C1	0.0328 (10)	0.0321 (11)	0.0272 (10)	0.0042 (9)	−0.0048 (8)	−0.0054 (9)
C2	0.0321 (11)	0.0369 (12)	0.0346 (11)	−0.0007 (9)	−0.0013 (9)	−0.0017 (10)
C3	0.0483 (13)	0.0303 (12)	0.0527 (14)	−0.0017 (10)	0.0010 (11)	−0.0058 (11)
C4	0.0546 (14)	0.0419 (14)	0.0519 (15)	0.0060 (11)	0.0082 (12)	−0.0167 (12)
C5	0.0532 (14)	0.0556 (16)	0.0409 (13)	0.0045 (12)	0.0173 (11)	−0.0064 (12)
C6	0.0462 (12)	0.0376 (13)	0.0361 (12)	−0.0007 (10)	0.0057 (10)	0.0002 (10)
C7	0.0451 (13)	0.0466 (14)	0.0474 (14)	−0.0053 (11)	0.0077 (10)	0.0029 (11)
C8	0.0316 (10)	0.0289 (11)	0.0278 (10)	−0.0013 (9)	0.0041 (8)	−0.0022 (8)
C9	0.0357 (11)	0.0322 (11)	0.0326 (12)	0.0035 (9)	0.0043 (9)	0.0022 (9)
C10	0.0503 (13)	0.0436 (14)	0.0429 (13)	0.0158 (11)	0.0041 (10)	−0.0044 (11)

### Geometric parameters (Å, º)

**Table:**

S1—C8	1.676 (2)	C4—C5	1.372 (3)
O1—C9	1.217 (2)	C5—C6	1.377 (3)
N1—C1	1.438 (3)	C9—C10	1.497 (3)
N1—C8	1.324 (2)	C3—H3	0.9300
N2—C8	1.388 (3)	C4—H4	0.9300
N2—C9	1.374 (3)	C5—H5	0.9300
N1—H1	0.8600	C6—H6	0.9300
N2—H2	0.8600	C7—H7A	0.9600
C1—C2	1.388 (3)	C7—H7B	0.9600
C1—C6	1.381 (3)	C7—H7C	0.9600
C2—C7	1.500 (3)	C10—H10A	0.9600
C2—C3	1.391 (3)	C10—H10B	0.9600
C3—C4	1.377 (3)	C10—H10C	0.9600
			
C1—N1—C8	123.96 (16)	O1—C9—N2	122.77 (19)
C8—N2—C9	128.34 (17)	C2—C3—H3	119.00
C1—N1—H1	118.00	C4—C3—H3	119.00
C8—N1—H1	118.00	C3—C4—H4	120.00
C8—N2—H2	116.00	C5—C4—H4	120.00
C9—N2—H2	116.00	C4—C5—H5	120.00
N1—C1—C2	120.27 (17)	C6—C5—H5	120.00
N1—C1—C6	117.88 (18)	C1—C6—H6	120.00
C2—C1—C6	121.80 (19)	C5—C6—H6	120.00
C3—C2—C7	121.32 (19)	C2—C7—H7A	109.00
C1—C2—C3	116.90 (18)	C2—C7—H7B	109.00
C1—C2—C7	121.79 (19)	C2—C7—H7C	109.00
C2—C3—C4	121.9 (2)	H7A—C7—H7B	109.00
C3—C4—C5	119.8 (2)	H7A—C7—H7C	109.00
C4—C5—C6	120.1 (2)	H7B—C7—H7C	109.00
C1—C6—C5	119.6 (2)	C9—C10—H10A	109.00
S1—C8—N1	124.12 (15)	C9—C10—H10B	109.00
S1—C8—N2	118.97 (14)	C9—C10—H10C	109.00
N1—C8—N2	116.91 (17)	H10A—C10—H10B	109.00
O1—C9—C10	122.74 (18)	H10A—C10—H10C	109.00
N2—C9—C10	114.49 (17)	H10B—C10—H10C	110.00
			
C8—N1—C1—C2	−79.9 (3)	C6—C1—C2—C3	−0.4 (3)
C8—N1—C1—C6	102.7 (2)	C6—C1—C2—C7	179.11 (19)
C1—N1—C8—S1	−0.9 (3)	N1—C1—C6—C5	177.42 (18)
C1—N1—C8—N2	179.42 (17)	C2—C1—C6—C5	0.1 (3)
C9—N2—C8—S1	177.45 (16)	C1—C2—C3—C4	0.8 (3)
C9—N2—C8—N1	−2.8 (3)	C7—C2—C3—C4	−178.76 (19)
C8—N2—C9—O1	5.5 (3)	C2—C3—C4—C5	−0.8 (3)
C8—N2—C9—C10	−175.01 (18)	C3—C4—C5—C6	0.4 (3)
N1—C1—C2—C3	−177.70 (17)	C4—C5—C6—C1	−0.1 (3)
N1—C1—C2—C7	1.8 (3)		

### Hydrogen-bond geometry (Å, º)

**Table:**

*D*—H···*A*	*D*—H	H···*A*	*D*···*A*	*D*—H···*A*
N1—H1···O1	0.86	1.99	2.664 (2)	135
N1—H1···O1^i^	0.86	2.50	3.172 (2)	135
N2—H2···S1^ii^	0.86	2.52	3.3747 (17)	171

Symmetry codes: (i) −*x*+2, −*y*, −*z*+1; (ii) −*x*+1, −*y*, −*z*.

## References

[bb1] Bernstein, J., Davis, R. E., Shimoni, L. & Chang, N.-L. (1995). *Angew. Chem. Int. Ed. Engl* **34**, 1555–1573.

[bb2] Bruker (2005). *SADABS* Bruker AXS Inc., Madison, Wisconsin, USA.

[bb3] Bruker (2009). *APEX2* and *SAINT* Bruker AXS Inc., Madison, Wisconsin, USA.

[bb4] Farrugia, L. J. (1997). *J. Appl. Cryst.* **30**, 565.

[bb5] Farrugia, L. J. (1999). *J. Appl. Cryst.* **32**, 837–838.

[bb6] Shahwar, D., Tahir, M. N., Chohan, M. M., Ahmad, N. & Samiullah (2012). *Acta Cryst.* E**68**, o508. 10.1107/S1600536812002371 PMC3275253 22347109

[bb7] Sheldrick, G. M. (2008). *Acta Cryst.* A**64**, 112–122. 10.1107/S0108767307043930 18156677

[bb8] Spek, A. L. (2009). *Acta Cryst.* D**65**, 148–155. 10.1107/S090744490804362X PMC2631630 19171970

